# The Association of Autoimmune Diseases with Type 1 Diabetes Mellitus in Children Depends Also by the Length of Partial Clinical Remission Phase (Honeymoon)

**DOI:** 10.1155/2020/2630827

**Published:** 2020-01-04

**Authors:** Gulsum Ozen, Angela Zanfardino, Santino Confetto, Alessia Piscopo, Francesca Casaburo, Nadia Tinto, Fernanda Iafusco, Gulsah Ozen, Emanuele Miraglia del Giudice, Medine Aysin Tasar, Arzu Yilmaz, Dario Iafusco

**Affiliations:** ^1^Department of Pediatrics, University of Health Science, Ankara Training and Research Hospital, Ankara, Turkey; ^2^Regional Centre for Pediatric Diabetes, Department of Pediatrics, University of Campania “Luigi Vanvitelli”, Naples, Italy; ^3^Department of Molecular Medicine and Biotechnology, CEINGE, Naples, Italy; ^4^Faculty of Medicine, Hacettepe University, Ankara, Turkey

## Abstract

Type 1 diabetes mellitus (DM) is characterized by irreversible, autoimmune, pancreatic *β*-cell destruction. During the disease, some patients experience a phase of Partial Clinical Remission (PCR) known as “*honeymoon*.” This is a transitory period that is characterized by insulin production by residual *β* cells following DM diagnosis and initiating the insulin therapy. In this study, we aimed to evaluate the influence of insulin production on immune system after the onset of diabetes, and we showed that the duration of *honeymoon* period could be related to the onset of other autoimmune conditions. For this retrospective study, 159 children aged between 11 and 18 years with type 1 DM were eligible. They have been diagnosed diabetes at least 10 years ago and use exogenous insulin. Our results showed that younger age at the onset of Type 1 DM in children, predicts Celiac Disease. Female sex and low HCO_3_ levels at the onset of DM had a high predictive value on patients who did not experience longer Partial Clinical Remission phase. Patients with higher BMI at the diagnosis of DM experienced shorter honeymoon period than the average. Smaller of our patients who diagnosed just DM have more than 297 days honeymoon period with respect to patients with one associated autoimmune disease. This may be due to a continuous and prolonged stimulation of immune system during the period of honeymoon that predispose the patient to develop other TH1 diseases. The patients who experienced more than 297 days Partial Clinical Remission seem under risk of developing one other autoimmune disease more than the patients who experienced less than 297 days Partial Clinical Remission. We have to consider that this observation is very intriguing because many protocols spring-up to try prolonging the honeymoon period in patients with autoimmune DM. If this aim is important from a metabolic point of view, long follow-ups are needed to be sure that the risk of other autoimmune diseases does not increase.

## 1. Introduction

Type 1 diabetes mellitus (DM) is a chronic illness known as insulin-dependent diabetes and characterized by irreversible, autoimmune, insulin producing islet *β*-cell destruction. Exogenous insulin administration is the only treatment for patients.

The cause of Type 1 diabetes mellitus is unknown, although genetic, immunologic, and environmental factors are known to increase the risk for its development. These suspected factors are mainly determined by the HLA genes which are located on chromosome 6, viral infections such as German measles, coxsackie and mumps, geography, family history, diet, stress events, perinatal factors, and other autoimmune conditions such as Hashimoto thyroiditis, multiple sclerosis, pernicious anemia, Sjogren's syndrome, idiopathic thrombocytopenic purpura, vitiligo, dermatitis herpetiformis, Addison's disease, and systemic lupus erythematosus.

Clinical symptoms of the disease arise after a significant reduction of *β-*cell mass when approximately less than 30% cells remain. However, patients retain some ability to regenerate *β* cells until decades after the clinical onset [1], although the autoimmune response destroys new *β* cells as they regenerate.

During the progression of the disease, some patients experience a phase of remission known as “honeymoon” or Partial Clinical Remission phase [2]. It is a transitory period that is marked by significant amount of endogenous insulin production by residual *β* cells following diabetes mellitus diagnosis and initiating the insulin therapy. During this phase, patients may require progressive smaller doses of insulin for good glycemic control. In literature, Partial Clinical Remission phase usually appears approximately 3 months after starting insulin therapy, but the duration ranges from 1 month [3] up to 13 years [4].

This special Partial Clinical Remission phase is a good model to study the mechanism of *β*-cell protection and may help the immunotherapy studies which aim to identify possible targets that may be used to cure type 1 diabetes mellitus or improve the disease's prognosis. Current research studies increasingly focus on the immunologic and metabolic factors to assess if they are influencing the remission rate and the duration of the honeymoon period. Recent studies have enlightened that an appropriate treatment and follow-up during the honeymoon could potentially enable the prolongation of this period for years or even permanently stop the destruction of the remaining *β* cells.

The patients who experienced Partial Clinical Remission phase or “*honeymoon*” period [5] have a significantly reduced risk of related long-term microvascular complications such as diabetic retinopathy, diabetic nephropathy, diabetic neuropathy, increase in statural growth in prepubescent children and reduced risk of severe hypoglycemia, but the prevalence of Partial Clinical Remission in children is approximately 50%, and this means that a significant proportion of children with type 1 Diabetes Mellitus will not experience Partial Clinical Remission.

In this study, we aimed to evaluate the influence of the residual production of insulin on immune system after the onset of diabetes and if the duration of *honeymoon* period could be related to the onset of other autoimmune conditions.

## 2. Material and Methods

The study was approved by the Ethics Committee of the University of Campania (Naples, Italy) and was conducted according to the Declaration of Helsinki. For this retrospective study, from 939 diabetic individuals with clinical data in the database “Aretheus” from Regional Centre for Pediatric Diabetes, University of Campania “L. Vanvitelli”—Naples, 159 children and adolescents aged between 11 and 18 years with type 1 diabetes mellitus (DM) were eligible for the inclusion. They have been diagnosed at least 10 years ago and use exogenous insulin therapy. Patients have been followed-up less than 10 years and have been excluded to give time for the expression of other autoimmune diseases associated with type 1 DM.

All the patients have been followed-up in the same centre and cured by the same medical team. This condition was ineluctable because the insulin dose may be influenced by different conditions and, most importantly, by the medical decisions.

The patients were divided into 2 groups: (1) patients with duration of honeymoon period less than 297 days and (2) patients with duration of honeymoon period more than or equal to 297 days. This number was the median of the duration of Partial Clinical Remission. Duration of honeymoon period has been compared with the number of autoimmune diseases developed.

The definition of the Partial Clinical Remission was normal glycemic values in the 24-hour profile together with daily insulin requirement <0.3°U/kg body weight/24 hours according to current Clinical Practice Guidelines [5].

The autoimmune diseases associated with DM that we have considered are: Hashimoto thyroiditis (HT), celiac disease (CD), and other autoimmune diseases (granuloma annulare (GA), rheumatoid arthritis (RA), vitiligo (Vt), Graves' disease (GD), and hereditary progressive spastic paraparesis (HPSP)).

Hashimoto thyroiditis was diagnosed by physical examination, laboratory testing of elevated thyroid stimulating hormone (TSH), and thyroid antibodies. The patients with elevated antibody levels were checked with thyroid USG. All patients diagnosed with Hashimoto thyroiditis had high level of TSH.

Celiac disease was diagnosed by elevated levels of certain antibodies and intestinal endoscopic biopsy.

Rheumatoid arthritis was diagnosed by physical examination and laboratory testing of serology.

Vitiligo, psoriasis and hereditary progressive spastic paraparesis were diagnosed by clinical examination.

We visited the patients there 1st week, 3rd week, 7th week, and every 3 month in each year after onset. The patients diagnosed with type 1 DM were examined at each control and practised autoimmune thyroiditis and celiac disease antibodies twice in a year in the first two years after DM diagnosis and then once a year.

The patients who had been diagnosed with type 1 DM more than 10 years ago were eligible for the inclusion. Although we have used automatic mechanical calculator and electronic patient diaries for 5 years, we did not have the possibility to calculate glycemic values automatically more than 10 years ago. In those days, the only method available in Italy was traditional paper diaries monthly. It was based on the data reported by patients. We accepted blood glucose level <180 mg/dl as a normal glycemic values in the 24-hour profile.

Statistical analysis were performed using SAS software version 9.4 (SAS Institute Inc., Cary, NC, USA), with a two-sided significance level <0.1. Descriptive statistics were used to summarize patient baseline characteristics, clinical data, and total insulin dose. Comparisons between the parameters were performed using *T* tests, Wilcoxon tests, or nonparametric Mann–Whitney tests for continuous variables, and Chi-squared tests or Fisher exact tests for extreme proportions for categorical variables.

## 3. Results

On a total of 159 patients (79 girls and 80 boys), 105 of them had just diabetes mellitus (66%), 48 of them had DM and one other autoimmune disease (30%), and 6 of them had DM and 2 other autoimmune disease related with DM (4%).

## 4. Discussion

This is the first study, to our knowledge, that has evaluated the duration of honeymoon in such a large number of pediatric patients with type 1 diabetes mellitus followed by the same diabetology team.

The definition of the Partial Clinical Remission was normal glycemic values (blood glucose < 180 mg/dl) in 24-hour profile together with daily insulin requirement <0.3 U/kg body weight/24 hours. Some of new studies defined the partial clinical remission as insulin-dose-adjusted haemoglobin A1c of ≤9. [6] In our study, having to give time to other autoimmune diseases to show up, we have chosen only the patients who had been diagnosed with type 1 DM more than 10 years ago. New definition was proposed 10 years ago (by Mortensen HB et al. in 2009) as insulin-dose-adjusted hemoglobin A1c of ≤9. But Lundberg RL et al. compared those two methods and did not see significant differences in the number of remitters, duration of Partial Clinical Remission, or the time of peak remission defined by IDAA1c of ≤9 or TDD of <0.3 units/kg/day [5].

Therefore, we do not think that defining the PCR as normal glycemic values in the 24-hour profile together with daily insulin requirement <0.3 U/kg body weight/24 hours will make a significant difference.

Our choice to carry out the study with patients of the same Pediatric Diabetology Centre has to be considered winning since the decision on the dose of insulin made by the same doctors can be decisive for evaluating Partial Clinical Remission.

The Partial Clinical Remission of diabetes mellitus depends essentially on the endogenous insulin production by residual *β* cells, and its length depends on the progressive destruction of these pancreatic *β* cells by the individual's immune system. If we consider patients with duration of honeymoon less than 297 days we have verified that a higher BMI determines a lower duration of the honeymoon period. This happens because the increase in BMI probably correlates with a greater insulin resistance that determines the need to increase the dose of insulin (Table 1).

In this study group, 8 hours fasting C-peptide levels were lower in patients with diabetes alone 0.39 ± 0.4 than in patients with diabetes + autoimmune diseases 0.53 ± 0.6 (*P* = 0.066).

Female sex, low HCO_3_ levels at the onset of DM had a high predictive value who did not experience a long Partial Clinical Remission phase (Table 2). This observation is in accordance with the literature data [7].

In this group of patients (159 patients), we had 26 patients with celiac disease. Twenty of them (77%) were diagnosed with DM before the age of 4. Six of them (23%) were diagnosed with DM after the age of 4 years. Also, 25 of them (96%) were diagnosed with DM before the age of 6 and 1 of them (4%) were diagnosed with DM after the age of 6. This observation confirms the data of Cerutti et al. [8] who observed that younger age at onset predicts Celiac Disease in children and adolescents with Type 1 Diabetes.

In our cases, the average duration of honeymoon (297 days) corresponds to that described in the literature which is 9.2 months (range 1.9–32.9 months) [9]. The results showed that as the duration of Partial Clinical Remission increases, there are more cases of association of Diabetes Mellitus with one other autoimmune disease with the TH1 mechanism (Table 3).

A small group of the patients who diagnosed with just DM have more than 297 days honeymoon period respect to patients with one other autoimmune disease associated. The prolonged production of insulin by the pancreatic *β* cells and continuous stimulation of the mediated T-lymphocyte immune system causes a longer duration of partial remission and could predispose the patient to develop other TH1 diseases (Table 4). Based on our recent knowledge, in the presence of other autoimmune diseases, the defect in the immune system will be more and different. It affects the prognosis of disease adversely. Besides that finding longer honeymoon periods does not give clear information about disease prognosis. Extended longitudinal analysis of larger cohorts will be needed to show the correlation between duration of honeymoon period and disease prognosis.

The mean of duration of Partial Clinical Remission in patients with diabetes alone was 713 days vs. 869 days in patients with diabetes and associated autoimmune disease(s) (*P* = 0.094).

The maximum amount of time for other autoimmune diseases to show up after diabetes mellitus was 3072 days (Hashimoto thyroiditis), so in our study, having to give time to autoimmune diseases to show up, we have chosen only the patients who had been diagnosed with DM more than 10 years ago. This has inevitably led to comparing patients who have not developed self-immunity after 10 years with patients who have not developed them after 17 years of follow-up. We do not have the possibility to fix this recruitment bias because if we had chosen only patients with an exact 10 year follow-up, the number of data would have been greatly reduced and we would never have reached statistical significance. On the other hand, in our series the average onset of autoimmune diseases is only 3.5 years, so it would not be a big mistake taking such a wide time window.

The mean occurrence time after DM diagnosis is 2 years for Celiac disease and 4 years for Hashimoto thyroiditis. It shows clinicians need to give heed to screening for related autoimmune diseases during these periods. The late onset of autoimmune disease from diabetes onset was after 3072 days for thyroiditis, 2768 for celiac disease (Table 5). This data is very interesting for the follow-up programming of patients with type 1 diabetes mellitus that can be reduced to 10 years with the certainty of diagnosing all associated autoimmune diseases [10].

We had just 1 case for each other autoimmune diseases except Hashimoto thyroiditis and celiac disease. They are not enough to compare the occurrence time with the onset of diabetes mellitus. Extended longitudinal analysis of larger cohorts will be needed to understand the relation of DM and other associated autoimmune diseases.

## 5. Conclusions

Our data show that the screening programming for detecting other autoimmune diseases is very critical in the first ten years after the onset of autoimmune diabetes and in particular children with DM age of onset younger than 4 years are at high risk of celiac disease.

The patients who experienced more than 297 days Partial Clinical Remission seem under risk of developing one other autoimmune disease associated with diabetes more than patients with less than 297 days Partial Clinical Remission. We have to consider that this observation is very intriguing because many protocols spring-up to try to prolong the honeymoon period in patients with type 1 DM. If this aim is important from a metabolic point of view, long follow-ups are needed to be sure that the risk of other autoimmune diseases does not increase.

## Figures and Tables

**Table 1:** Characteristics of study population at enrolment.

	Mean	Girls	Boys
Age (years) ± SD	15.51 ± 1.9	15.5 ± 1.8	15.52 ± 2
Body mass index (BMI) (kg/m^2^)	18.94	18.12	19.35
Weight percentile (%)	54.98	50.27	59.68
8 hours fasting C-peptide level (ng/ml)	0.43	0.46	0.4
Age on onset (years)	3.55	3.51	3.59
Duration of diabetes mellitus (years)	12.33	12.39	12.33
	Range: 10.28–17.2	Range: 10.30–17.23	Range: 10.31–15.94

**Table 2:** Comparison of characteristics of patients divided for partial clinical remission duration.

	Partial clinical remission < 297 days	Partial clinical remission ≥ 297 days	*P* value
Age (years)	15.6	15.4	*P* = 279 NS
Gender (%)	M: 36 F: 64	M: 58 F: 42	χ square 6–405 DF = 1 *P* = 0.011
HCO_3_^−^ at onset (mg/dl)	18.7	19.3	*T* = −1.323 *P* = 0.0188
BMI (kg/m_2_)	20.7	17.6	*P* = 0.01
Duration of DM (years)	12.1	12.4	*T* = −0–574 *P* = 0.566 NS

The mean duration of honeymoon phase was 297 days as correlated with literature (an average is 9.2 months). In the study population; female sex, low HCO_3_ levels at the onset of DM had a high predictive value on patients who did not experience more than 297 days Partial Clinical Remission.

**Table 3:** Prevalence of other autoimmune diseases associated with diabetes mellitus in patients without, with less and more than 297 days partial clinical remission.

	Without partial clinical remission	Partial clinical Remission <297 days	Partial clinical Remission ≥ 297 days
Diabetes mellitus	26 (74%)	11 (61%)	68 (64%)
Diabetes mellitus + 1 other autoimmune disease	8 (23%)	5 (28%)	35 (33%)
Diabetes mellitus + 2 other autoimmune diseases	1 (3%)	2 (11%)	3 (3%)

Eight (23%) of the patients who did not experience a phase of Partial Clinical Remission had been followed-up with DM and 1 other autoimmune disease. Five (28%) of the patients who experienced less than 297 days, a phase of Partial Clinical Remission had been followed-up with DM and 1 other autoimmune disease. Thirty-five (33%) of the patients who experienced more than 297 days a phase of Partial Clinical Remission had been followed-up with DM and 1 other autoimmune disease. This results show that clinicians should be more careful for the other autoimmune diseases, with the patients who experience longer Partial Clinical Remission.

**Table 4:** Prevalence of honeymoon periods according to patients diagnosed with just diabetes mellitus, one other autoimmune disease, and two or more other autoimmune diseases.

	Without partial clinical remission	Partial clinical remission <297 days	Partial clinical remission ≥297 days
Diabetes mellitus	26 (25%)	11 (10%)	68 (65%)
Diabetes Mellitus + 1 other autoimmune disease	8 (17%)	5 (10%)	35 (72%)
Diabetes mellitus + 2 other autoimmune diseases	1 (17%)	2 (33%)	3 (50%)

105 patients were diagnosed with just diabetes mellitus. 26 of them (25%) did not experience a phase of partial clinical remission. 11 of them (10%) experienced less than 297 days, and 68 of them (65%) experienced more than 297 days Partial Clinical Remission phase. 48 patients were diagnosed with Diabetes Mellitus and 1 other autoimmune disease. 8 of them (17%) did not experience a phase of partial clinical remission. 5 of them (10%) experienced less than 297 days and 35 of them (72%) experienced more than 297 days Partial Clinical Remission phase. 6 patients were diagnosed with diabetes mellitus and 2 other autoimmune diseases. 1 of them (17%) did not experience a phase of partial clinical remission. 2 of them (33%) experienced less than 297 days, and 3 of them (50%) experienced more than 297 days Partial Clinical Remission phase.

**Table 5:** Occurrence times for other autoimmune diseases related to the onset of diabetes mellitus.

	Minimum	Maximum	Mean
Diabetes mellitus + celiac disease (26 patients)	64	2768	800
Diabetes mellitus + hashimoto thyroiditis (29 patients)	27	3072	1382
Diabetes mellitus + granuloma annulare (1 patient)	498	498	498
Diabetes mellitus + rheumatoid arthritis (1 patient)	−1297	−1297	−1297
Diabetes mellitus + hereditary progressive spastic paraparesis (1 patient)	1020	1020	1020
Diabetes mellitus + graves' disease (1 patient)	−408	−408	−408
Diabetes mellitus + vitiligo (1 patient)	2032	2032	2032

26 patients were followed-up with celiac diseases. Occurrence time after DM diagnosis for celiac disease is minimum 64 days and maximum 2768 days. Mean occurrence time for these 26 patients is 800 days. 29 patients were followed-up with Hashimoto thyroiditis. Occurrence time after DM diagnosis for Hashimoto thyroiditis is minimum 27 days and maximum 3072 days. Mean occurrence time for these 29 patients is 1382 days. 1 Rheumatoid arthritis and 1 Graves' disease were diagnosed before DM diagnosis. Celiac disease comes up about 2 years after the onset of DM and Hashimoto thyroiditis comes up about 4 years after the onset of DM.

## Data Availability

All the original data used to support the findings of this study have been deposited in the software “ARETHEUS” property of the Regional Centre of Pediatric Diabetology “G. Stoppoloni” of the University of Campania “Luigi Vanvitelli,” Naples, Italy.
